# Emotional Intelligence among Nurses and Its Relationship with Their Performance and Work Engagement: A Cross-Sectional Study

**DOI:** 10.1155/2023/5543299

**Published:** 2023-11-10

**Authors:** Fatimah Turjuman, Bayan Alilyyani

**Affiliations:** ^1^Madinah Cardiac Center, Ministry of Heath, Medina, P.O. Box 42351, Saudi Arabia; ^2^Department of Nursing Management and Education, College of Nursing, Taif University, 2425 Taif, Saudi Arabia

## Abstract

**Background:**

Several studies identified that emotional intelligence skills are important indicators for nurse engagement and performance. Issues related to nursing performance in healthcare organizations have been gaining greater attention because they influence the effectiveness of improvement approaches to maintain high-quality care. This study aimed to investigate the relationships between emotional intelligence and nurses' work performance and work engagement.

**Methods:**

A quantitative, descriptive, correlational design was used to evaluate the relationships between the study variables. Data were gathered from 150 nurses working at Madinah Cardiac Center, Saudi Arabia. Three scales were used to measure the study variables which were Emotional Intelligence Scale, Job Performance Scale, and Utrecht Work Engagement Scale in addition to demographics. SPSS was used to analyze data.

**Results:**

The results of this study showed that emotional intelligence has a total mean of 3.77 (SD = 0.598), nurses' performance 3.65 (SD = 0.503), and work engagement 4.29 (SD = 1.04). The results also showed that there is a positive and significant relationship between emotional intelligence and nurses' work performance (*R*^2^ = 0.657, *p* < 0.001). Also, it was found that emotional intelligence has a positive and significant relationship with nurses' work engagement (*R*^2^ = 0.621, *p* < 0.001).

**Conclusions:**

This paper highlights the influence of emotional intelligence in nurses' improved performance and engagement in work. The field of nursing is associated with care and compassion; thus, it needs a high level of emotional intelligence. Nurses need to enhance their emotional intelligence skills by attending workshops. Nurse leaders also have a role in that by building a culture for nurses that is driven by applying emotional intelligence in the workplaces.

## 1. Introduction

Nurses' roles are versatile from promoting and restoring patients' health to overseeing the quality of care and supervising nursing teams [1]. Dealing with different duties and responsibilities under different conditions on the daily basis requires nurses to enhance personal and professional skills in healthcare settings. In healthcare, professional nursing practice relies heavily on organizational and personal engagement [2]. Nursing staff engagement is not only a vital variable for quality care [3] but also a source of personal satisfaction and wellbeing in clinical practice [4]. Employees who are engaged in their work accept any recommendations related to their profession and are loyal to their organizations where they work at [5]. Studies showed that work engagement among nurses is associated with different factors such as work environment [6], optimism, and self-efficacy [7]. On the other hand, emotional intelligence (EI) skills, such as self-awareness, emotion control, motivation, and recognition, were found as important indicators for nurse engagement and performance [8]. Furthermore, enhancing EI skills positively relates to personal satisfaction, wellbeing, and, ultimately, positive nursing outcomes [9]. Nurses' work engagement is significantly predicted by the EI resources in workplace [4]. There is a previous study conducted to examine the relationship between emotional intelligence and work engagement among nurses working in critical areas and it found that there is a significant positive correlation between emotional intelligence and work engagement [10].

EI as a developmental tool is beneficial in maintaining nurses' ability to carry on with their jobs; however, other aspects related to the workplace, such as support from the supervisor or training on empathy in patient care, can be associated with work performance [4]. Job preference of workers depends on different factors including goals and values of each organization [11]. Given the unique challenges facing healthcare professionals after the recent pandemic, it is very important for nurses to enhance their EI skills, such as self-motivation as it has a significant impact on their performance [8]. Nurses who lack of emotional intelligence often find it difficult to handle work pressure, and consequently, their work performance goes down. Having the necessary EI skills can allow nurses to foster and nurture their emotional and interpersonal potential, which can be turned into useful tools to overcome challenges in the workplace [9]. For example, in the context of patient-centred care, practicing clinical empathy reflects the nurse's ability to recognize different emotions and respond to their patient with appropriate emotion.

Further, EI also enables nurses to grasp the idea of healthcare as a complex system. This can be particularly important for nurses working in the Saudi healthcare system, which is going under fundamental reforms and requires interdisciplinary collaboration to embrace the new improvement approaches [12]. In the current context, issues related to nursing performance (e.g., skills, competencies, motivation) in healthcare organizations have been gaining greater attention because they influence the effectiveness of improvement approaches to maintain high quality of care [12]. Understanding the influence of EI in nursing practice can shed light on ways to improve nursing engagement and performance. Having the necessary emotional intelligence could allow nurses to handle any situations during a specific period of time because they are able to use their interpersonal relationships and their previous experiences [13]. This allows nurses to provide the needed and safe care for each patient in their workplaces [8]. Further, having emotional intelligence also plays a very significant role in the nurses being able to have self-motivation and self-confidence [14]. This eventually results in creating a better and more cohesive work environment for everyone, especially the nurses [14]. Moreover, around 60–70% of nurses working in Saudi hospitals are non-Saudi who had different cultures, nationalities, and backgrounds [15] which may affect their emotional intelligence.

This paper aimed to investigate the relationships between emotional intelligence, performance, and work engagement among nurses at the Madinah Cardiac Center. This study is significant as it analyzes and formulates the importance of emotional intelligence in improving nurse's job performance and work engagement in the organization by examining their relationships. The research on this matter thus brings to light the importance of emotional intelligence in relation to the work engagement and performance of nurses in the Medina Cardiac Center. It also highlights the role of the organization in providing the appropriate support and resources to foster and nurture EI in nursing practice and achieve healthcare organization goals.

### 1.1. Theoretical Framework

Emotional intelligence is defined as the ability of an individual to apply their emotions as an effective tool in their social environment [16]. People with high level of emotional intelligence are those who know themselves very well which also helps to know the emotions of others [17]. Emotional intelligence was introduced firstly by Salovey and Mayer [18] and later by Goleman [19] via his book, Emotional Intelligence: Why It Can Matter More than IQ. They described emotionally intelligent individuals as those are able to monitor their minds and others [19]. They are also strong, sociable, and believing positive about others [17]. There are five main domains of emotional intelligence that cover both personal and social aspects which are self-awareness, self-regulation, self-motivation, social awareness, and social skills [19].

Work engagement is defined by Schaufeli et al. [20] as “… a positive, fulfilling, work-related state of mind that is characterized by vigor, dedication, and absorption” (p. 74). Job performance is defined as the expectations behaviours of individuals regarding organization's goals and values [21].

### 1.2. Research Hypotheses

**Figure 1:**
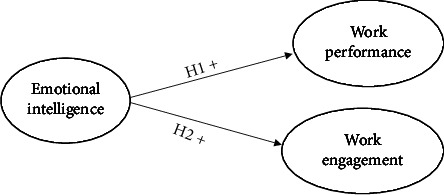
Conceptual framework.

Emotional intelligence incorporates self-awareness to nurses to understand patients' feelings and emotions. It assists them in structuring patients' expectations. In this way, nurses gain insights to take actions accordingly. The aspects of emotional intelligence including self-awareness, self-regulation, self-motivation, social awareness, and social skills help to improve job performance of staff [22]. They also are essential elements for enhancing their work engagement [22].  Figure 1 illustrates the conceptual framework. Thus, the following hypotheses were proposed:

 
*H*1: there is a positive relationship between emotional intelligence and job performance.

Increased emotional intelligence includes elements such as motivation and empathy. Self-motivation of nurses drives the achievement of goals personally and is helpful in nursing leadership as well. At the same time, empathy builds strong connections between nurses and patients. The feeling of shared trust increases loyalty towards the organization and secures work engagement, thus a positive relationship is built coming out from emotional intelligence influencing the work engagement of nurses [23]. Thus, the following hypothesis was proposed:

 
*H*2: there is a positive relationship between emotional intelligence and work engagement.

## 2. Materials and Methods

### 2.1. Design

This research followed a quantitative, descriptive, correlational design to examine the relationships between the study variables which are emotional intelligence, performance, and work engagement. This design is the most suitable design for this study because it examines the relationships between study variables [24].

### 2.2. Setting

Data were collected from inpatient departments in Madinah Cardiac Center including Emergency Department (ED), Intensive Care Unit (ICU), Cardiac Care Unit (CCU), and medical, surgical, and outpatient clinics during the months of June–September 2022. The center is located in Madinah city in Saudi Arabia. This center is operated by Saudi Ministry of Health (MOH). It has 100-bed capacity. The center focuses on treating all cardiac-related problems.

### 2.3. Sample and Inclusion Criteria

In this research, convenience sampling was applied because it was the method for this research, and researchers did not have access to whole list of nurses working in the center. The number of participants were 150 nurses from the various departments of Madinah Cardiac Center. The questionnaire was distributed to approximately 385 nurses, and 150 responses were received. The target sample was 193 nurses. The sample size was calculated by using the confidence level 95% and a margin of error 5. All nursing staff, both male and female, registered nurses, were able to complete the surveys in English and assigned in inpatient departments (ED, ICU, CCU, medical, surgical) and outpatient clinics were included in the study. However, non-nursing staff, nonregistered nurses, were not assigned in inpatient departments (ED, ICU, CCU, medical, surgical) and outpatient clinics were excluded.

### 2.4. Instruments

The instrumentation included multiple questionnaires relevant to the previously conducted scientific studies. Three surveys were used to measure the study variables in addition to demographics.

Emotional intelligence was measured by using the self-report scale [25]. It consists of 33 items based on 5 subscales: internal motivation (7 items), self-regulation (7 items), self-awareness (7 items), empathy (6 items), and social awareness (6 items). Items are rated on a five-point Likert scale ranging from 1 (Completely Disagree) to 5 (Strongly Agree). Validity and reliability values of the scale were measured in the previous studies [26, 27].

Nurses' performance was measured by using a five-item self-rating scale [28]. It consists of total 9 items with two subscales. The subscale contains professional development (5 items) and evaluation of care (4 items). The items are rated on a five-point Likert scale ranging from 1 (Strongly Disagree) to 5 (Strongly Agree). Psychometric analysis including validity and reliability values was examined in the previous studies [28, 29].

Work engagement was measured by using The Utrecht Work Engagement Scale (UWES) [30]. It consists of 17 items based on three subscales: vigor (6 items), dedication (5 items), and absorption (6 items). The items are rated on a seven-point Likert Scale ranging from 0 (never) to 6 (always). Previous studies measured the validity and reliability of the scale [30, 31].

Demographics were measured by investigating multiple questions which included (gender, nationality, age, educational qualifications, work experience, and working department).

### 2.5. Data Collection Plan

Data were collected from the nursing staff at Madinah Cardiac Center after obtaining ethical approval. Administration of Education Department coordinated with the nurse leaders and notified them about the study. Then, electronic questionnaires were sent to the Education Department, which distributed the link of questionnaires to departments of the center.

### 2.6. Data Analysis

In this research, SPSS was used to analyze data. Descriptive analysis was used to analyze demographic and Cronbach's alpha to measure the reliability of main study variables scales. Regression and correlation were applied to analyze the relationships between main study variables.

### 2.7. Ethical Consideration

The approval was obtained from the Administration of Education Department in Madinah Cardiac Center to collect the research data, and decision was issued on June 1, 2022. Participants were asked a question about their agreements to voluntary participation in the study before completing the surveys as informed consent. Also, submitting a complete survey means they agreed to participate in the study as they were informed in the information letter. There were no identifications collected from the participants to ensure privacy. Also, participants had the right to withdraw from the study any time. All information was kept in a locked file, and only researchers can access them.

## 3. Results

### 3.1. Analysis of Demographics

The analysis of demographics is explained in Table 1. The demographic analysis reveals that most of the respondents had 5 years of experience in the role of a nurse. Almost 60% respondents were within the age group of 26–30 and 31–35 years of age group. Thus, it can be seen that most respondents are from the young age group who have a long job life to peruse. But another 37% of the respondents were from the age group of above 35 years and therefore are capable of providing mature opinion. Most of the nurses were female 87.3% and had a Bachelor's degree (84%). Majority of the respondents were non-Saudi (66%). Most of the respondents were from the “Intensive Care Unit” (23.3%). Table 1 illustrates the demographics of participants.

### 3.2. Descriptive Statistics and Reliability of Main Study Variables

The descriptive and reliability of the main study variables are explained in Table 2. All three scales used in this study showed acceptable results of Cronbach's alpha ranged between 0.635 and 0.786. As shown in Table 2, emotional intelligence has a total mean of 3.77 (SD = 0.598) with Cronbach's alpha of 0.635. Nurses' performance has a total mean of 3.65 (SD = 0.503) with Cronbach's alpha of 0.699. Finally, work engagement has a total mean of 4.29 (SD = 1.04) with Cronbach's alpha of 0.786.

### 3.3. Relationships between Demographics and Main Study Variables

The relationships between demographics and main study variables are explained in Table 3. The results showed that there are significant relationships between emotional intelligence and gender (*t* = 2.07, *p*=0.039), nationality (*t* = 3.10, *p*=0.002), and department where nurses work in (*F* = 2.28, *p*=0.039). In addition, there are significant relationships between nurses' performance and gender (*t* = 2.33, *p*=0.021), nationality (*t* = 2.61, *p*=0.010), and age (*F* = 2.63, *p*=0.052). For work engagement, it was found that work engagement has significant relationships with nationality (*t* = 3.30, *p*=0.001), level of education (*F* = 3.52, *p*=0.032), and work experience (*F* = 2.82, *p*=0.041).

### 3.4. Relationships among Study Variables

The relationships among study variables are explained in Table 4. The regression analysis showed that emotional intelligence has a positive and significant effect on nurses' performance (*R*^2^ = 0.657, *p* < 0.001). Also, emotional intelligence was found to have a positive and significant influence on nurses' work engagement (*R*^2^ = 0.621, *p* < 0.001). The correlations among the study variables are illustrated in Table 5.

## 4. Discussion

This study aimed to examine the relationships between emotional intelligence and performance and work engagement of the nurses at Madinah Cardiac Center. The findings of this study showed that emotional intelligence is strongly associated with nurses' work performance and indicated that if the emotional intelligence of a nurse is high enough, then the work performance of that nurse is supposed to be high because the value of the correlation coefficient is not only positive but also quite a high score. Also, it indicated that emotional intelligence and work performance are moving in the same direction. This result supported a previous study conducted by Geun and Eunok [4] who found that emotional intelligence plays a vital role in execution of a work. Also, nurses who are emotionally intelligent are more capable to execute the work efficiently [4]. It is true that there are other factors which could affect nurses' performance such as support from the supervisor, empathetic capability, and self-motivation, but emotional intelligence is of highest influence over the working performance of a nurse [4]. Thus, nurses with high emotional intelligence are more capable to handle stress while providing care for their patients [32]. Another study found the same results of the current study by linking the emotional intelligence with job performance of nurses [8]. A study was conducted in Saudi Arabia by Alsufyani et al. [33] to examine the relationship between EI and work performance among nurses and found that emotional intelligence and work performance are positively associated. Nurses with a high level of emotional intelligence are capable to handle work which results in better overall work performance [14]. According to Alonazi [8], without a good level of emotional intelligence, nurses will find it difficult to handle the day-to-day stress and work pressure which in turns could affect their performances and increase the pore performance in comparison to the nurses who are having a higher level of emotional intelligence and comparatively better work performance. Therefore, it is very essential that emotionally intelligence must be present among the nurses as it could enable them to adjust with any situations and enhance patient's outcomes [8].

The results of this study also showed that emotional intelligence and work engagement are positively and significantly related. This indicated that a nurse who is emotionally intelligent has a higher degree of engagement to the work. This result is consistent with a previous study conducted to examine the connection between emotional intelligence and work engagement and found that emotional intelligence is significantly and positively connected with work engagement [10]. The results supported that emotional intelligence improved work engagement [2]. Another study aimed to explore the impact of emotional intelligence on work engagement among psychiatric care nurses and found that emotional intelligence enhances work engagement of nurses [34]. Other previous studies also supported the results of the current study by finding that emotional intelligence directly influences nurses' work engagement [35, 36]. Moreover, in a recent study that was conducted to assess the role of work engagement as a mediator between emotional intelligence and work performance among healthcare workers, it was found that emotional intelligence was positively related to both work engagement and work performance, and work engagement meditated the relationship between emotional intelligence and work performance [37].

In other words, emotionally intelligent nurses should be involved with their own work, and this not only enhances their work performance but also enhances their level of engagement [34]. The nurse who is most engaged with their work is supposed to stay in the organization for a longer period [38]. The correlation results also came up with another crucial insight which showed that there is a high positive correlation between the work engagement and work performance. This indicated that nurses who have a strong engagement in their work are definitely going to perform in a better manner than other nurses who are having less engagement towards their work [39].

Nurses should be aware of how emotional intelligence could affect their work and the care that they deliver to their patients. Previous studies showed that some factors have an impact on emotional intelligence among nurses such as supervisor support, proper work environment, self-motivation ability, and educational programs [32, 40]. In this scenario, nurse leaders should ensure that the work environment is conducive in order to provide the best care for each patient [32]. Further, there should be educational programs that developed in healthcare organizations with the focus on ensuring that nurses are able to have better emotional control and are able to deal with different situations wherein they have to exercise a lot of emotional control while executing their work [32].

### 4.1. Implications

Our results can be implemented and beneficial for nursing field in general and nursing practice in particular as well as nursing research. Emotional intelligence should be applied by nurses in their practices by being aware of their emotions and managing them. Also, nurses can apply emotional intelligence when interacting with their colleagues and patients. Nurses need to make a balance when using emotions in their practices. This helps them to take the right action at the right time to meet the unique needs of each patient. Nurses should be emotionally intelligent by paying attention to self-awareness and managing emotions in order to enhance their engagement and performance which in turn helps to achieve organization goals and improve the healthcare system. As previously identified, there are limited studies conducted that explore the effect of emotional intelligence in nursing in Saudi Arabia. Therefore, further research should be undertaken to explore the relationship between emotional intelligence and nurses and patients' outcomes. Also, more studies are needed that should focus on exploring ways to enhance nurses' emotional intelligence.

### 4.2. Limitations

Although the significance of the results is showed in this study, it also has some limitations. The small sample size was a limitation of this study resulting from poor response rate from the participants. Also, the use of cross-sectional design prevented the causal relationship to be explored in this study. Limiting the study to nursing staff worked in one healthcare center affects the generalizability of the study results to not be applied to all nurses working in Saudi Arabia. There is a possibility for bias when participants may not provide accurate information as a result of social desirability bias.

## 5. Conclusions

This study revealed the existence of a positive relationship between emotional intelligence and job performance as well as a positive relationship between emotional intelligence and work engagement among nurses working at Madinah Cardiac Center in Saudi Arabia. Thus, emotional intelligence appears as the main driving factor behind the improvement of the overall working quality of those nurses. This study emphasizes the positive impact and tangible difference can be made in the nursing field as a result of using emotional intelligence; this effect will not be limited only on the nurses, but it will include patients as well and even the health facility outcomes.

## Figures and Tables

**Table 1:** Demographic characteristics.

Demographics	Number	Percent
*Age*		
20–25	5	3.3
26–30	42	28.0
31–35	47	31.3
Above 35	56	37.3

*Gender*		
Female	131	87.3
Male	19	12.7

*Educational level*		
Bachelor's degree	126	84.0
Diploma degree	11	7.3
Master's degree	13	8.7

*Nationality*		
Non-Saudi	99	66.0
Saudi	51	34.0

*Working experience*		
1–3 years	17	11.3
4-5 years	18	12.0
Less than 1 year	5	3.3
More than 5 years	110	73.4

*Department*		
Cardiac Care Unit	32	21.3
Emergency Department	9	6.0
Intensive Care Unit	35	23.3
Medical Ward	7	4.7
Others	52	34.7
Outpatient Department	2	1.3
Surgical Ward	13	8.7

**Table 2:** Descriptive statistics and reliability of main study variables.

Variables	Number of items	Mean	Std. Dev	Cronbach's alpha
Emotional intelligence	33	3.77	0.598	0.635
Nurses' performance	9	3.65	0.503	0.699
Work engagement	17	4.29	1.04	0.786

**Table 3:** Relationships between demographics and main study variables.

Variables	Demographics					
	Gender	Nationality	Age	Educational level	Work experience	Department
Emotional intelligence	*p*=0.03988, *t* = 2.07	*p*=0.00235, *t* = 3.10	*p*=0.16822, *F* = 1.71	*p*=0.09620, *F* = 2.38	*p*=0.23857, *F* = 1.56	*p*=0.03905, *F* = 2.28
Nurses' performance	*p*=0.02120, *t* = 2.33	*p*=0.01011, *t* = 2.61	*p*=0.05262, *F* = 2.63	*p*=0.35329, *F* = 1.05	*p*=0.20193, *F* = 1.56	*p*=0.24887, *F* = 1.33
Work engagement	*p*=0.12690, *t* = 1.54	*p*=0.00122, *t* = 3.30	*p*=0.08111, *F* = 2.29	*p*=0.03200, *F* = 3.52	*p*=0.04116, *F* = 2.82	*p*=0.05762, *F* = 2.09

*Note*. ^*∗*^*p* < 0.05.

**Table 4:** Relationships among study variables.

Study variables	*R* ^2^	*F*	d*f*	*p* value
Emotional intelligence and nurses' performance	0.657	112	d*f*1 = 1	<0.00001
			d*f*2 = 148	

Emotional intelligence and work engagement	0.621	92.7	d*f*1 = 1	<0.00001
			d*f*2 = 148	

^*∗*^ *Note*. *p* < 0.001.

**Table 5:** Correlation matrix among study variables.

	EI	*P*	*W*
EI	—	—	—
*P*	0.657^*∗∗∗*^	—	—
*W*	0.621^*∗∗∗*^	0.591^*∗∗∗*^	—

*Note*. ^*∗*^*p* < 0.05, ^*∗∗*^*p* < 0.01, and ^*∗∗∗*^*p* < 0.001. EI, emotional intelligence; *P,* performance; *W*, work engagement.

## Data Availability

Access to data is restricted due to ethical concerns and privacy of participant information.
